# The National Deficit of Black and Hispanic Physicians in the US and Projected Estimates of Time to Correction

**DOI:** 10.1001/jamanetworkopen.2022.15485

**Published:** 2022-06-01

**Authors:** Hector Mora, Adetokunbo Obayemi, Kevin Holcomb, Maurice Hinson

**Affiliations:** 1 Weill Cornell Medical College, New York, New York; 2 Black and Latino Men in Medicine, NewYork-Presbyterian Weill Cornell Medical Center, New York, New York; 3 Hospital of the University of Pennsylvania, Philadelphia; 4 NewYork-Presbyterian Weill Cornell Medical Center, New York, New York; 5 SUNY Upstate Medical University Hospital, Syracuse, New York; 6 NewYork-Presbyterian Queens, Queens, New York

## Abstract

This cross-sectional study of US population and physician workforce data projects rates at which medical school matriculation would have to increase for Black and Hispanic medical students to reach equal representation in the workforce.

## Introduction

The Association of American Medical Colleges (AAMC) projects a total physician racial and ethnic representation deficit of between 37 800 and 124 000 physicians by 2034.^1,2^ Recent work has found that while the “numbers and proportions of Black, Hispanic, and American Indian or Alaska Native medical school matriculants [have] increased, [they have done so] at a rate slower than their age-matched counterparts in the US population, resulting in increased underrepresentation.”^3^ These disparities in representation at the medical school level portend worsening disparities at the physician workforce level, and could worsen the already dire racial and ethnic disparities in outcomes such as infant and maternal mortality, obesity, and life expectancy.^4^ A 2019 study by Lett et al^3^ notes, “Given the duration of physician training, any changes implemented [to improve recruitment and retention of students from underrepresented racial and ethnic minority groups] will take more than a decade to affect the physician workforce.” This cross-sectional study compares the self-reported demographics of the US population with the demographics of the US physician workforce and estimates the time it would take to reach a representative population of Black and Hispanic physicians.

## Methods

In this cross-sectional study, we compared the demographics of the US population with the US physician workforce for the years 2010 and 2015.^5^ Since data from both the US Census Bureau and AAMC are publicly available, aggregated, and deidentified, the study qualified for exemption from Weill Cornell Medical College institutional review board approval and informed consent requirements.

Assuming a physician workforce with equal representation of racial and ethnic groups seen in the US population during the years of 2010 and 2015, as defined by the US Census Bureau,^6^ we calculated the relative overrepresentation and underrepresentation of physicians for each racial or ethnic group in both years (ie, American Indian, Asian, Black or African American, Hispanic, Pacific Islander, and White). We then calculated how long it would take to achieve a representative physician workforce if a sustained doubling, tripling, or quadrupling of the number of Black or Hispanic allopathic medical student matriculants were to take effect from 2015. This cross-sectional study adheres to the Strengthening the Reporting of Observational Studies in Epidemiology (STROBE) reporting guideline. Analysis was conducted using Excel version 2203 (Microsoft).

## Results

**Figure 1.**
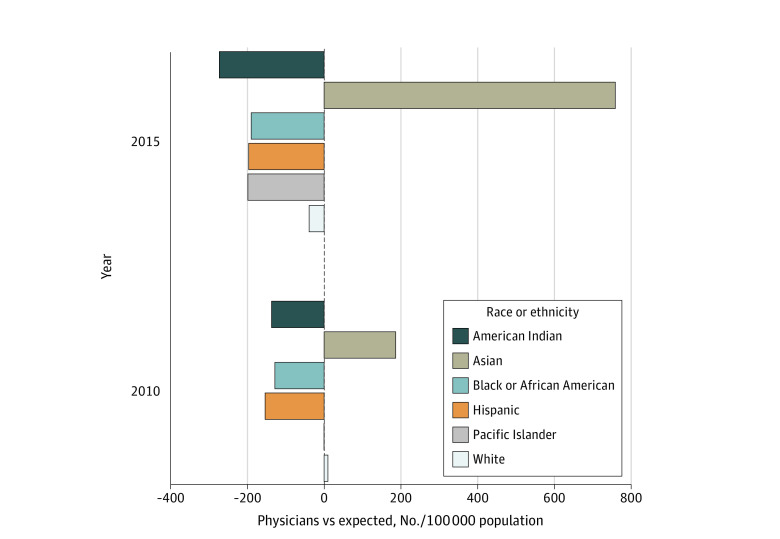
Representation of Physician Workforce Relative to US Population per 100 000 From 2010 to 2015 Data for Active Pacific Islander physicians were unavailable for 2010.

**Figure 2.**
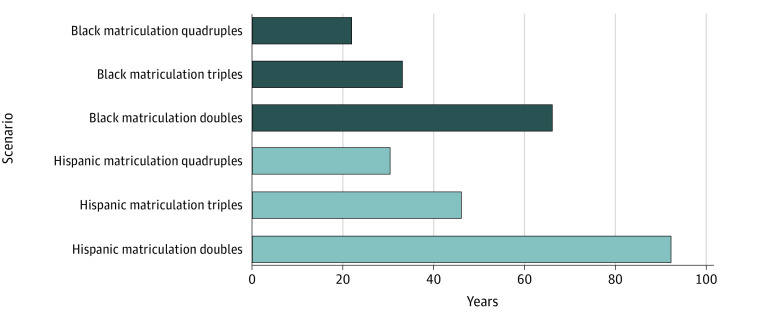
Years to Correct Black and Hispanic Physician Deficits in Different Scenarios Where the Annual Matriculation Doubles, Triples, or Quadruples

In 2015, there were 20 349 allopathic medical school matriculants and 961 098 practicing physicians of all races and ethnicities. Of those, there were 1231 Hispanic and 1228 Black medical students. Concurrently, there were 60 549 Hispanic and 46 133 Black physicians. Based on their representation in the population, the expected numbers would be 174 307 Hispanic and 127 490 Black physicians. Thus, we calculated a deficit of 113 758 Hispanic and 81 358 Black physicians. Compared with the US population, there were 196 and 191 fewer Hispanic and Black physicians, respectively, per 100 000 Hispanic and Black people in the US (Figure 1). Given these estimates, it would take 92 years of a sustained doubling of the number of matriculating Hispanic medical students in 2015 to correct the deficit of Hispanic physicians from 2015. Meanwhile, it would take 66 years of a sustained doubling of Black medical students to correct the deficit of Black physicians from 2015 (Figure 2).

## Discussion

According to the American Medical Association,^1^ racial and ethnic diversity among health professionals promotes better access to health care, improves health care quality for underserved populations, and better meets the health care needs of our increasingly diverse population. If the goal is to achieve a diverse and representative physician workforce within our lifetimes, a sustained and multifaceted approach must be implemented that will address both the size of the underrepresented medical school applicant pool as well as the number of underrepresented medical students and postgraduate trainees. Given AAMC’s projected physician shortage of roughly 37 800 to 124 000 physicians by 2034,^2^ the creation and expansion of medical schools that prioritize the education of Black, Hispanic, and other underrepresented students would not only decrease the overall physician shortage, but also shorten the time required to attain a representative physician workforce and help mitigate the societal harm inflicted by decades of structural racism.

Our estimations were limited by not accounting for future trends in national demographics or immigration, which could underestimate the time to reach a representative Black and Hispanic physician workforce. Additionally, we did not account for possible changes in the number of medical school and residency training positions available in the US, nor did we have access to more granular data on racial and ethnic groups, such as by nationality or ancestry.
